# Correction: Wu et al. Rust Fungi on Medicinal Plants in Guizhou Province with Descriptions of Three New Species. *J. Fungi* 2023, *9*, 953

**DOI:** 10.3390/jof9111068

**Published:** 2023-11-01

**Authors:** Qianzhen Wu, Minghui He, Tiezhi Liu, Hongmin Hu, Lili Liu, Peng Zhao, Qirui Li

**Affiliations:** 1 State Key Laboratory of Functions and Applications of Medicinal Plants, Guizhou Medical University, Guiyang 550004, China; wqz1665@aliyun.com (Q.W.); minghuihegmu1979@163.com (M.H.); a2942338310@aliyun.com (H.H.); lililiu550025@163.com (L.L.); 2 The High Efficacy Application of Natural Medicinal Resources Engineering Center of Guizhou Province (The Key Laboratory of Optimal Utilization of Natural Medicine Resources), School of Pharmaceutical Sciences, Guizhou Medical University, University Town, Guian New District, Guizhou 550004, China; 3 College of Chemistry and Life Sciences, Chifeng University, Chifeng 024000, China; tiezhiliu@aliyun.com; 4 Immune Cells and Antibody Engineering Research Center of Guizhou Province, Guizhou Medical University, Guiyang 550004, China; 5 Key Laboratory of Biology and Medical Engineering, Guizhou Medical University, Guiyang 550004, China; 6 State Key Laboratory of Mycology, Institute of Microbiology, Chinese Academy of Sciences (CAS), Beijing 100101, China

**Error in Figure**

In the original publication [1], there were two mistakes in Figure 3 as published. The names of two new species were incorrect. The correct name is *Phragmidium cymosum*, while the incorrect name is *Phragmidium cymosa*. Additionally, the correct fungal name is *Hamaspora rubi-alceifolii*, while the incorrect name is *Hamaspora alceaefolius*. The corrected Figure 3 appears below. The authors state that the scientific conclusions are unaffected. This correction was approved by the Academic Editor. The original publication has also been updated.

## Figures and Tables

**Figure 3:**
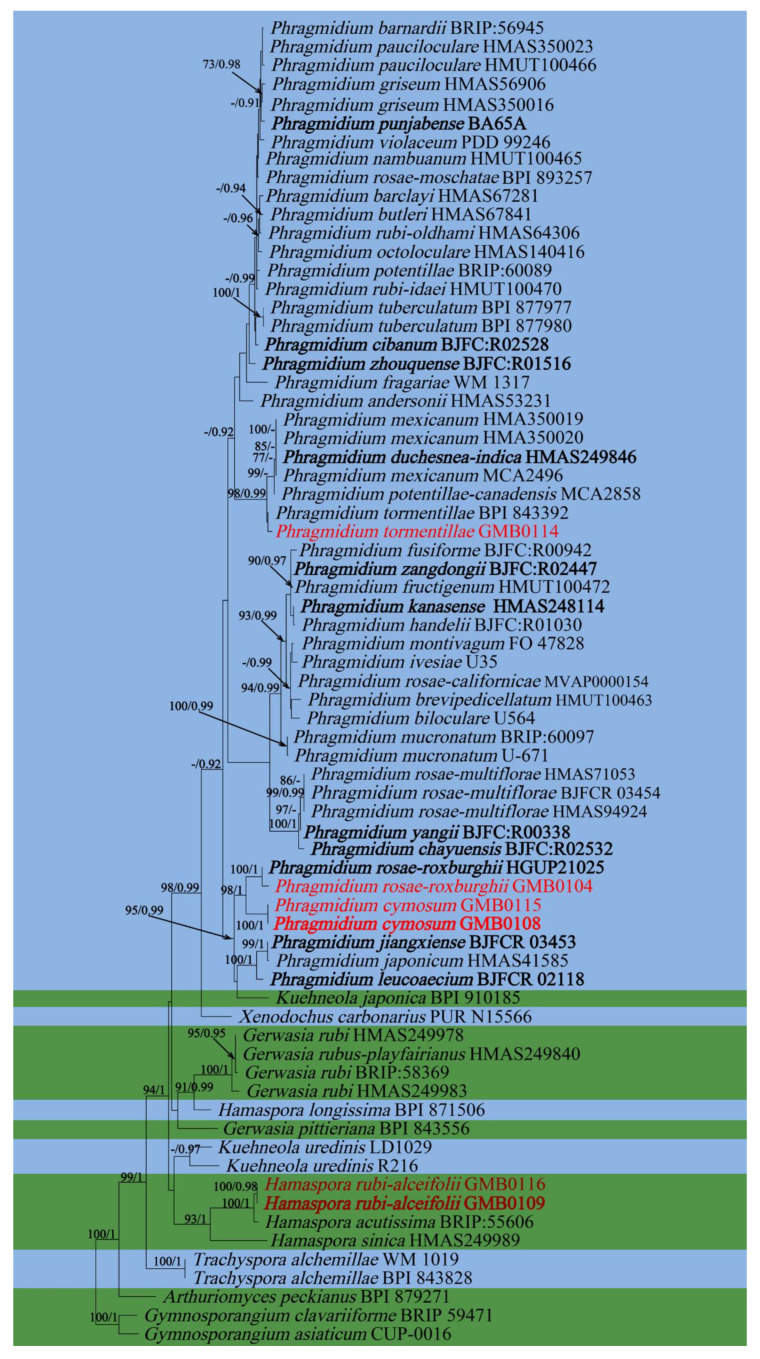
RAxML tree of the family *Phragmidiaceae* based on rDNA ITS and LSU sequences. ML bootstrap supports (≥70%) and Bayesian posterior probability (≥0.90) are indicated as ML/BYPP. The tree is rooted to *G. asiaticum* and *G. clavariiforme* [11]. The type specimens are shown as boldface. New sequences are in red.
